# Lecythophora Soft-Tissue Infection: Case Report and Treatment Considerations

**DOI:** 10.7759/cureus.42919

**Published:** 2023-08-03

**Authors:** Ryan Golden, David S Quimby

**Affiliations:** 1 Infectious Diseases, CommonSpirit Health, Omaha, USA; 2 Medicine/Infectious Diseases, Creighton University, Omaha, USA

**Keywords:** infection, tenosynovitis, fungal, terbinafine, lecythophora

## Abstract

*Lecythophora hoffmannii* is a saprophytic fungus commonly found in the environment. Able to be isolated from soil, it is frequently associated with the soft rot of wood. Although human infections are not common, they have been reported, and have ranged from keratitis and soft-tissue infection to deep osteomyelitis and endometritis. Here we report a case of soft-tissue infection with this pathogen along with successful treatment with standard-dose terbinafine when other agents were unavailable. The true prevalence of infections with this pathogen is unclear and further data are needed to determine optimal therapy.

## Introduction

*Lecythophora (Coniochaieta) hoffmannii* is a saprophytic fungus commonly found in the environment and associated with soft rot of wood [1]. Infections due to this pathogen are relatively rare in medical literature, but cases of keratitis, sinusitis, peritonitis, soft-tissue infections, osteomyelitis, and endometritis have been documented [1-6]. As with many emerging pathogens, immunocompromised hosts may be particularly at risk. The current prevalence of infection from this fungal pathogen is unknown and may be underreported. Given the relative paucity of published information, optimal treatment strategies are also lacking. Here, we report a case of soft-tissue *L. hoffmannii* infection managed with terbinafine.

## Case presentation

**Figure 1:**
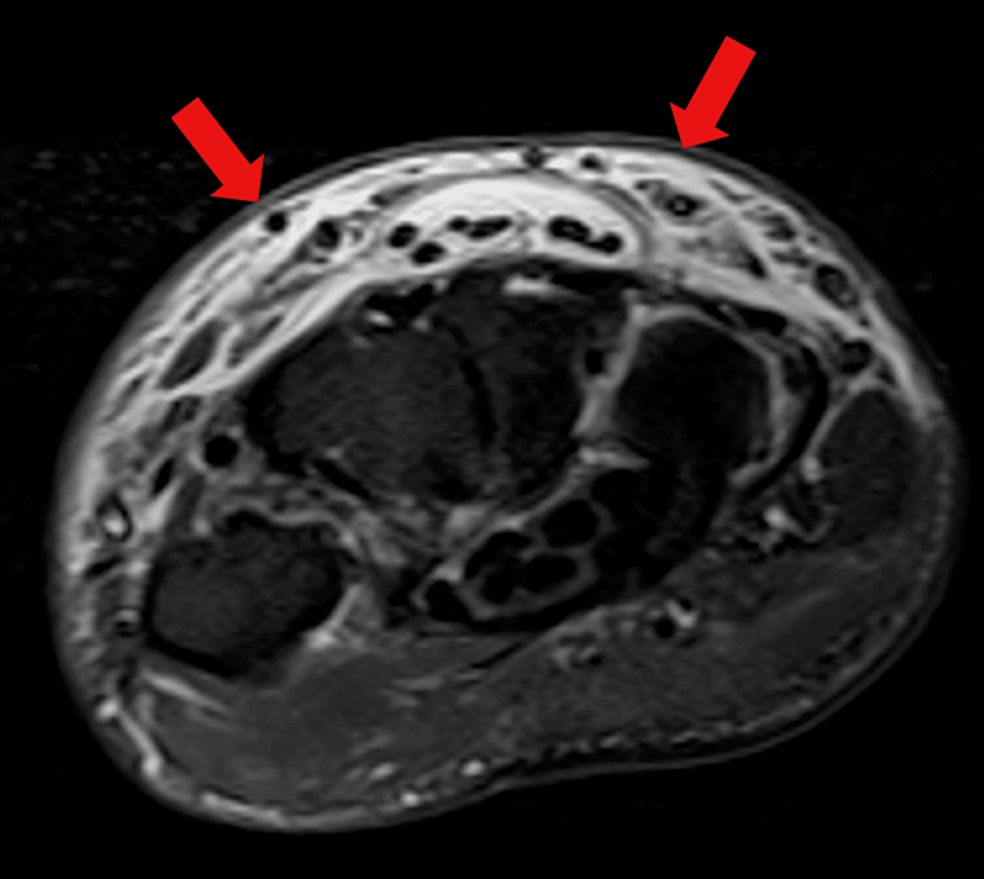
MRI axial STIR image showing dorsal edema STIR: Short Tau Inversion Recovery

**Figure 2:**
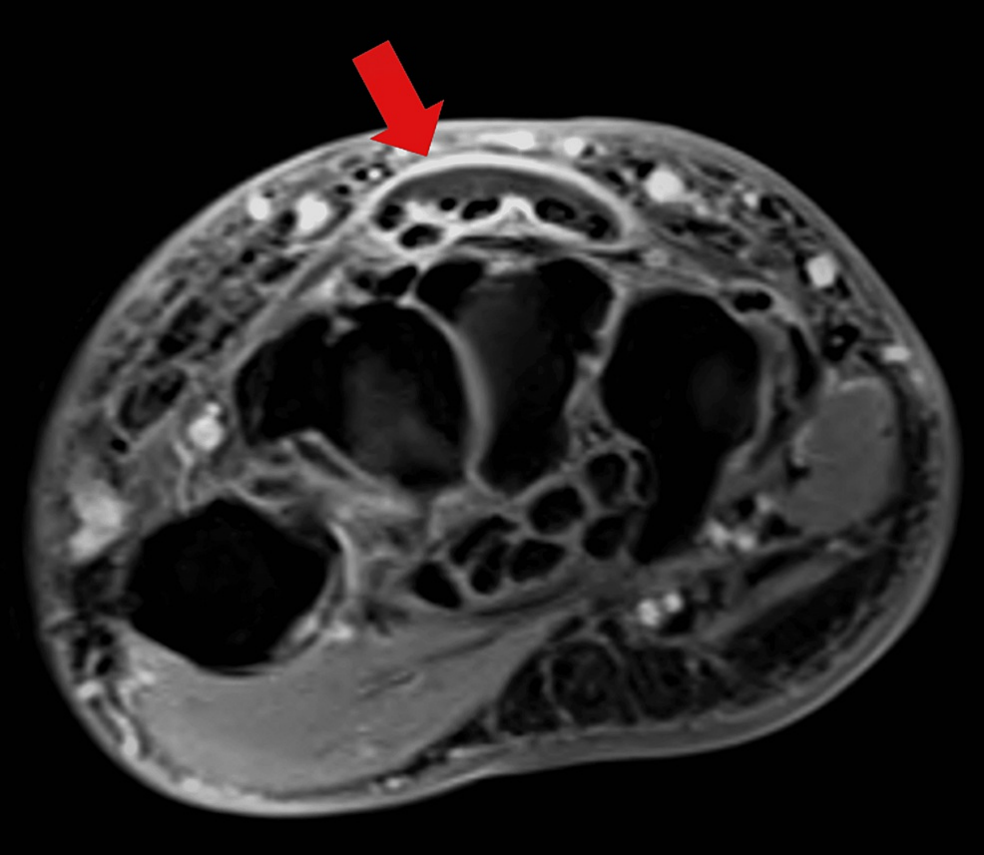
MRI axial T1 image post-contrast showing enhancement of and fluid within tendon sheaths

A 75-year-old man with a medical history most relevant for inflammatory arthritis and Crohn's disease was working on the retaining wall of his landscaping pond during the summer when he felt a sharp puncture-type feeling in his forearm. His inflammatory arthritis was managed with metrotrexate for two years and sulfasalazine for greater than five years; the inflammatory bowel disease was managed with vedolizumab for three years following prior use of certolizumab for five years. The following morning, he had swelling of the arm from the elbow to the metacarpophalangeal joints and he presented to the hospital. He was afebrile without signs or symptoms of systemic illness. On examination, there was a small puncture wound on the dorsal surface of the forearm with mild surrounding erythema along with diffuse swelling. His range of motion for finger flexion as well as hand supination/pronation was limited due to both pain and swelling. Plain radiographs showed no bony abnormalities. An MRI was obtained, showing soft-tissue swelling (Figure 1), extensor digitorum tenosynovitis (Figure 2), and a small radiocarpal-joint effusion.

**Figure 3:**
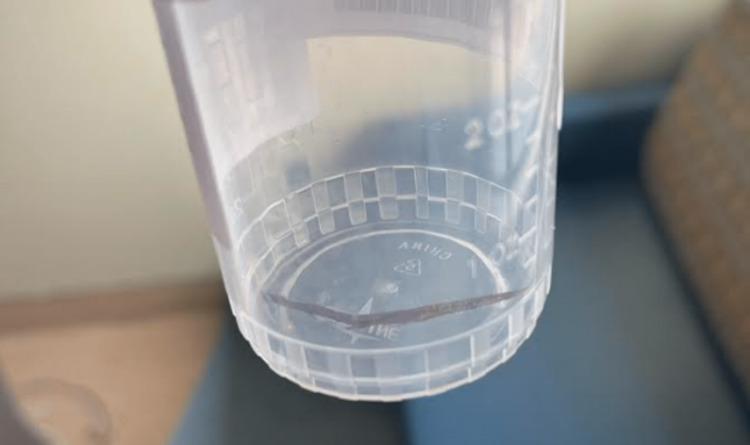
Foreign body removed at the time of surgery

He was initially treated with vancomycin, piperacillin-tazobactam, and clindamycin. The following day, he still had significant pain and a diminished range of motion. Aspiration of the wrist yielded purulent appearing fluid. He underwent operative exploration later that day. No purulence was noted at the time of surgery, but there was extensive inflammation and a wooden foreign body (approximately 2mm x 3mm x 30mm, Figure 3) was removed from the soft tissues; following removal, he underwent pulsatile irrigation. Intraoperative cultures were not obtained. No organisms were seen on the pre-operative aspiration Gram stain. Due to ongoing symptoms, he underwent a second irrigation two days after the first; again no purulence was seen. As bacterial cultures were negative, he was dismissed on an empiric antibacterial regimen of amoxicillin-clavulanate and doxycycline for presumptive infectious tenosynovitis.

**Figure 4:**
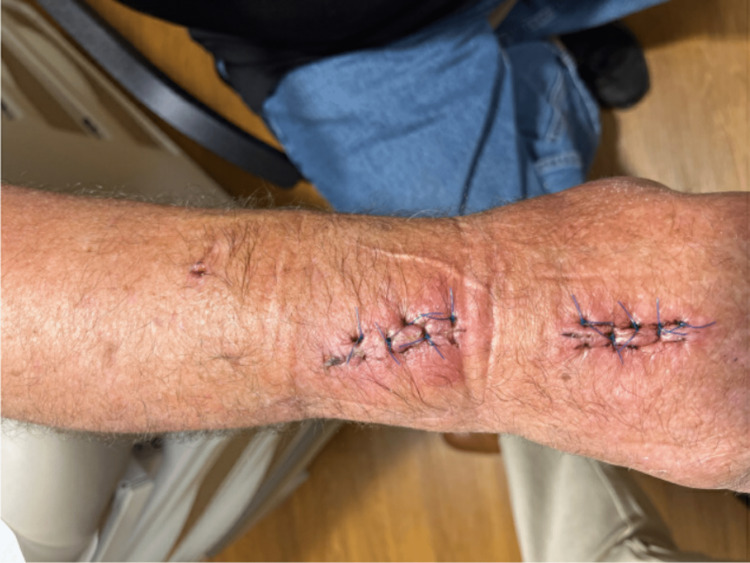
Wrist appearance on postoperative day eight

**Figure 5:**
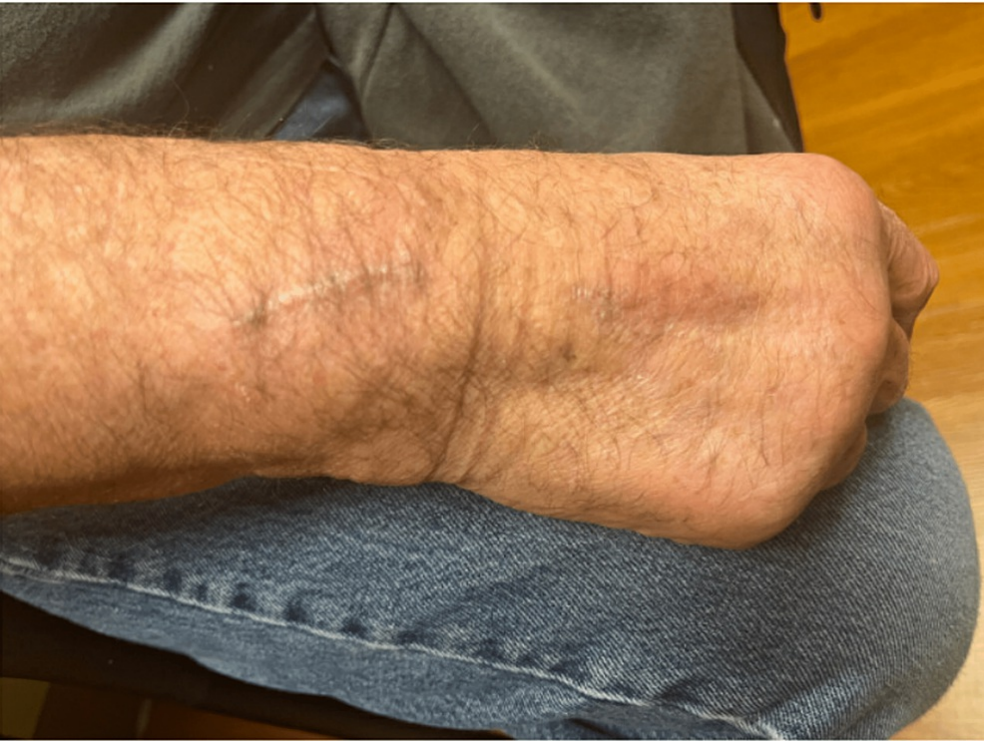
Wrist appearance on postoperative day 59

After hospital discharge, fungal growth was noted in the aspiration culture. This was identified as *Lecythophora hoffmannii *via mass spectroscopy; *Penicillium* spp. was later identified as well. On postoperative day (POD) eight, he was clinically doing fair with expected postoperative swelling about his proximal incision (Figure 4); he still had some pain to palpation of the general area. Given his immunocompromised state, the decision was made to treat the positive fungal culture even though he was having clinical improvement at the time. He was prescribed posaconazole. Unfortunately, there were insurance-related barriers to his obtaining the medication. By POD ten, he was noting an increase in both pain and hand swelling. Given his inability to obtain posaconazole, an attempt was made to prescribe him isavuconazole while appeals were in process for the posaconazole. Unfortunately, he was not able to obtain this agent either. By POD 15, the hand was continuing to worsen, and the patient was prescribed terbinafine 250mg daily while the ongoing appeals for other antifungal agents were in progress. By the fourth day of terbinafine therapy, he noted that his hand swelling and pain started to improve. By POD 26 (the twelfth day of terbinafine therapy), he noted that swelling and range of motion were markedly improved. Examination on POD 59 (Figure 5) showed a full range of motion, no pain or erythema, and only mild residual swelling. He completed three months of terbinafine and has had no issues with the hand or wrist after completion of antifungal therapy.

## Discussion

The nomenclature of this organism has changed over the years. Initially classified as *Margarinomyces hoffmannii*, it was later changed to *Phialophora hoffmannii*, followed by *Lecythophora hoffmannii* and the currently accepted *Coniochaieta hoffmannii* [1,7]. The genus *Lecythophora* is still frequently used in clinical literature.

Perdomo et al. obtained eight samples of *L.hoffmannii* as part of their study of *Phialemonium* and *Lecythophora* clinical isolates [4]. Minimum inhibitory concentrations (MICs) were determined for these isolates; itraconazole, posaconazole, and voriconazole had clinically acceptable results [4]. This is in contrast to the susceptibility results in some case reports [5,8] which suggested much higher MICs for the azole antifungals. Given our inability to obtain posaconazole or isavuconazole, along with the terbinafine MICs noted in the Perdomo study [4], terbinafine was used in our patient with good clinical outcome. Antimicrobial susceptibility testing was unable to be performed on our isolates.

Published literature may underrepresent the frequency of infection with *Lecythophora* spp. Despite only a handful of publications with these pathogens, Perdomo et al. were able to identify multiple isolates for their investigation [4]. If infections with this genus are more prevalent than suspected, further data collection regarding susceptibility will be important for future management when this pathogen is identified. The optimal duration of therapy is also very unclear given the paucity of published data. Finally, another area ripe for investigation would be the optimal dosing of older agents such as terbinafine. When used for attempted synergy for non-onychomycosis infections, higher doses are frequently used, which can lead to higher plasma levels of the medication [9]. The role of such dosing for soft tissue infection such as in our patient, however, is unclear given the relatively high drug levels historically obtained in the epidermal tissue when examined [10].

In our case, two fungi were identified, *Lecythophora hoffmannii* and *Penicillium* spp. It is impossible to determine the relative contribution of each to our patient's infection. *Penicillium *spp are ubiquitous environmental organisms usually involved with pulmonary or disseminated infection in immunocompromised patients, but rare cases of cutaneous infection can be found in the literature [11]. When tested, this genus frequently shows in vitro susceptibility to terbinafine [12].

## Conclusions

*Lecythophora hoffmannii* is a fairly ubiquitous environmental organism but seems underreported in the medical literature. There are limited case reports and series about infection with this pathogen, but optimal therapy (choice of antifungal agent and duration) remains unclear. Although the newer azole antifungals show both *in vitro* and clinical efficacy against this organism, this case demonstrates that the older antifungal agent terbinafine may also be effective. As the identification of unusual fungal pathogens such as *Lecythophora* may be increased with the use of newer diagnostic techniques such as mass spectroscopy in clinical laboratories, there may be increasing data in the literature in the future to help guide clinicians to therapeutic options.
